# A case report of unilateral cervical lymphadenopathy and multiple cranial neuropathies following mRNA-COVID-19 vaccination

**DOI:** 10.1186/s12883-022-02900-1

**Published:** 2022-09-26

**Authors:** Fatma Shalabi, Alexander Lossos, Dimitrios Karussis

**Affiliations:** Department of Neurology, Hadassah Medical Organization and Faculty of Medicine, Hebrew University, Jerusalem, Israel

**Keywords:** Case report, Messenger RNA vaccine, COVID-19, Cranial Neuropathy, Lymphadenopathy

## Abstract

**Background:**

We report a rare case of ipsilateral multiple cranial neuropathy and ipsilateral lymphadenopathy following mRNA-COVID-19 vaccination.

**Case Presentation:**

A 41-year-old male visited our emergency room complaining of dysphagia and hoarseness that started a week after receiving COVID19 mRNA vaccination (in his right arm). During his hospitalization, he also complained of right side hearing loss and diplopia. Neurological examination depicted a right IV nerve palsy, ipsilateral facial paresthesia and peripheral facial paresis. Otorinolaryngological examination revealed right vocal cord paralysis. A brain magnetic resonance imaging showed enhancement of the right VII and VIII cranial nerves in the auditory canal. The lumbar puncture revealed increased protein concentration and lymphocytic pleocytosis in the cerebrospinal fluid (CSF). Additionally, a neck computed tomography (CT) scan showed a swollen right supraclavicular lymph node. We hypothesize that the ipsilateral cranial neuropathies of IV, VI, VII, VIII and X, associated with cervical lymphadenopathy, was possible caused by a post-vaccination immune-mediated reaction. The patient was treated with a 5-day course of intravenous methylprednisolone (1000 mg/day), and a gradual improvement was observed.

**Conclusions:**

Similarly, to other vaccines, it is possibly that also mRNA vaccines may act as triggers of non-specific autoimmune neurological syndromes.

## Background

Various neurological syndromes have been reported in association with vaccines, including cranial nerve (CN) palsies [1]. A wide range of putative immunological pathogenic mechanisms have been suggested [1], but the exact relationship of these syndromes with the vaccinations, remains highly controversial. Vaccines in general, may act as triggers for autoimmune reactions, since they contain both infectious agents or their immunogenic proteins and chemical adjuvants, cumulatively acting as triggers of immune system activation ([2], CDC 24/7), that involves humoral, cellular immunity and inflammatory cytokines’ release.

The newly introduced messenger-RNA vaccines (Pfizer-BioNTech and Moderna) for COVID-19, utilize a novel technology, using lipid nanoparticles formulated in nucleoside-modified mRNA that encodes the prefusion spike glycoprotein of COVID-19, aiming to produce spike proteins by the recipient cells after the entrance of the mRNA into their cytoplasm and subsequently an anti-spike protein immune response [3]. Such mRNA vaccines were shown in the past to induce a strong generalized activation of the immune system [4]. We hereby report a case of an individual, who developed unilateral cranial neuropathy and cervical lymphadenopathy proximal to the site of vaccination, a week after the first dose of COVID19 mRNA-vaccination.

## Case presentation

A previously healthy 41-year-old male was evaluated in our Neurological Department due to subacute dysphagia evolving over 5 days. The patient also complained of dizziness, hoarseness and pain on the right shoulder, radiating to the neck, a week after COVID-19 mRNA-vaccination (Pfizer-BioNTech BNT162b2), which he received in his right arm. The next few days, he developed right hearing loss and right facial weakness. Neurological examination revealed mild hoarseness, right facial paresthesia, a mild right peripheral facial palsy and hypoactive gag reflex, other CN was intact. No other focal signs were detected, including normal motor, sensory and cerebral function, normal deep tendon reflexes, normal gait and no long tract signs.

**Fig. 1:**
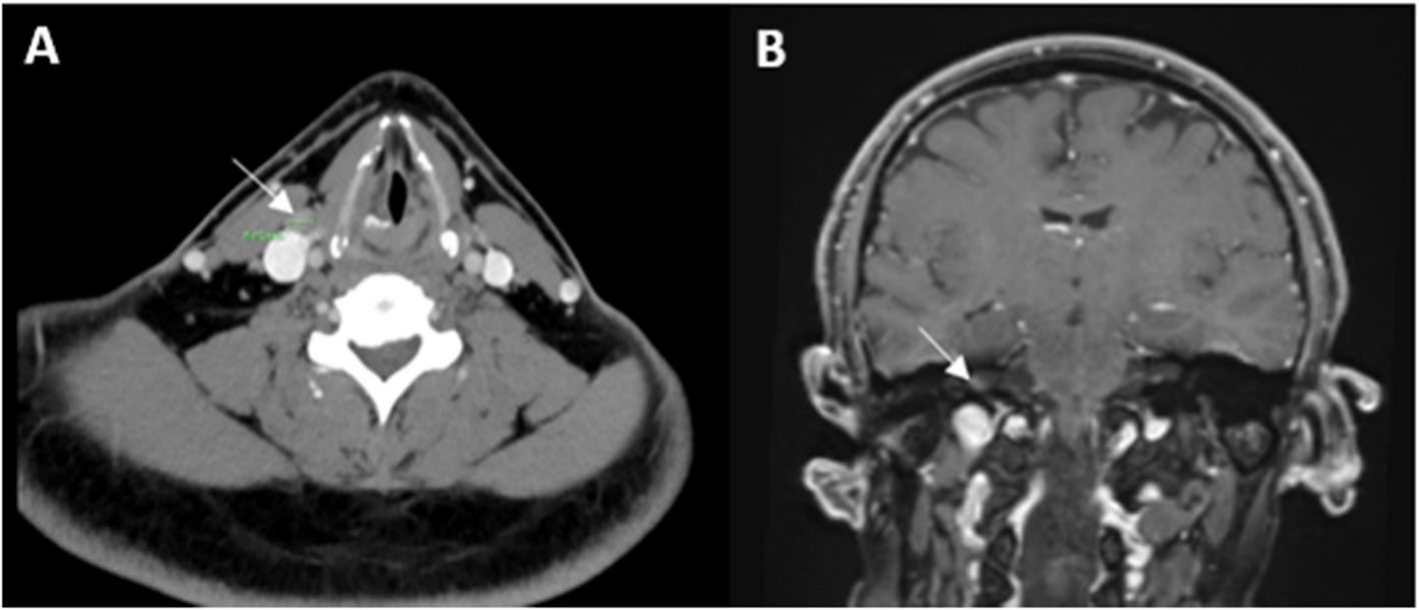
**A** Axial neck CT image demonstrating a 1 cm right supraclavicular reactive lymph node (arrow). **B** Coronial brain (with Gadolinium) MRI image showing enhancement in the right auditory canal, suggesting involvement of Cranial nerves VII and VIII (arrow)

Otolaryngological examination diagnosed a right vocal cord paralysis. A chest and neck CT scan showed thickening of the right vocal cord and a swollen right supraclavicular lymph node (LN) (Fig. 1A). An audiogram depicted right severe high-frequency (HF) mixed hearing impairment and left HF sensorineural hearing impairment.

Later on, the patient started complaining of vertical diplopia, mainly downwards and to the left side, indicative of a right IV^th^ nerve palsy, with no other new neurological focal signs. A brain Magnetic resonance imaging (MRI) showed enhancement in the right auditory canal, which involved the VII^th^ and VIII^th^ CNs (Fig. 1B). Fast Imaging Employing Steady-state Acquisition FIESTA-sequence MRI did not depict involvement of other CNs or other pathology. The lumbar puncture revealed increased protein (691 mg/l) and normal glucose (3.4 mmcl/l) concentrations and CSF leukocytosis (63 cells/ml). Cytopathology of the CSF showed two different types of complete B-cell rearrangement with low intensity polyclonal background without evidence of atypical or tumor cells. CSF immune-electrophoresis did not detect intrathecal oligoclonal antibodies. Routine blood tests and an extensive panel of autoantibodies (including ANA, ANCA, and anti-gangliosides) were within normal ranges. Tumor markers and a paraneoplastic antibodies panel (in serum and CSF) were negative. Polymerase chain reaction for Herpes simplex virus (1 and 2), Human herpesvirus (HHV) 3, HHV-6, Enterovirus, JC virus and Cytomegalovirus were negative. Cryptococcal antigen and direct acid fast bacilli staining in of the CSF, nasopharyngeal swab for COVID19, serology for Human immunodeficiency virus and Syphilis were also negative. A total body CT ruled out systemic neoplasia. The titers of anti-COVID19 spike protein antibodies were high (IgG: 441 AU/ML; pos. > 50 AU/ML).

After ruling out all the above causes, our working hypothesis was that of an immune mediated multiple cranial polyneuropathy and a 5-day course of intravenous methylprednisolone at 1000 mg/day was administered. Under this treatment, a gradual significant improvement was observed in all symptoms and findings. A follow up audiogram showed significant improvement and a neck ultrasound revealed resolution of the swollen LN.

## Conclusions

Various immune mediated neurological complications following vaccinations have been reported [1]. Post-vaccination CN palsies are rare. In a review summarizing all motor CNs palsies other than VII, following various vaccines, the CN III, IV and VI were the most frequently involved [1]; However, no case of unilateral multiple cranial neuropathy was included in this series.

Axial lymphadenopathy related to COVID-19 mRNA-vaccination (particularly BNT162b2) has been reported by the FDA Briefing Document. In our patient, the proximity of the lymphadenopathy ipsilateral to the site of vaccination in the arm and the close temporal relation of the clinical onset of the neurological syndrome, raise the possibility that the vaccine served as trigger of an immune-mediated cascade that involved several CNs (IV, V, VII, VIII and X, but not VI, although it was reported to be common). The inflammatory nature of the process is supported by the high protein levels and the lymphocytosis in the CSF, and the finding of two different types of complete B-cell rearrangements, indicating the presence of immunoreactive lymphocytes and/or antibodies in the CSF.

Concerning the possible immunopathogenesis of this syndrome, we hypothesize that activated lymphocytes may have been generated in the local LNs at the lymphatic drainage area of the arm and migrated through the blood or lymphatic circulation to the brainstem, causing an inflammatory process in the subarachnoid space surrounding the lower CNs, ipsilaterally. The possible mechanism of such immune-mediated process targeting the CNs, could be related to cross-reactivity of the produced anti-spike antibodies (and lymphocytes) with CNS antigens due to molecular mimicry. Such cross-reactivity with a number of autoantigens (including CNS antigens, as myelin basic protein) has been reported [5]. Widespread bystander activation of the immune system following COVID19-vaccination could provide an alternative explanation for the pathogenesis of this syndrome, and might be supported by the finding of the non- specific B-cell rearrangements in the CSF. The rapid improvement of symptoms and the resolution of the LN swelling after steroid treatment, further support the inflammatory immune-mediated nature of the neurological syndrome.

The differential diagnosis includes, other causes of basal meningeal inflammation, such as sarcoidosis, Behcet’s disease, neurolymphomatosis, IgG4-related disease or histiocytosis. The absence of a clonal population on immunophenotyping of CSF and of any other peripheral immune deviation, make these possibilities unlikely but, in some cases, only histo-pathological examination can completely rule out such possibilities. The clinical picture and the presence of albimunocytologic dissociation could be supportive of Guillain Barre Syndrome or Miller Fisher syndrome. However, the normal deep tendon reflexes and the absence of any other focal signs, along with the extreme asymmetry of the findings, make this possibility rather unlikely, although an atypical variant of GBS cannot be ruled out.

Finally, the possibility of random co-occurrence of the syndrome with COVID-vaccination cannot be totally excluded (despite the close temporal association of the events), especially since COVID vaccination is now highly prevalent across the developed world.

In summary, the presented case raises the possibility that COVID-19 mRNA-vaccination, similarly to other vaccines, may act as trigger of immune-mediated syndromes through non-specific activation of the immune system, molecular mimicry or bystander effects. However, this case still presents an isolated and probably rare event and given the documented efficacy and general safety profile of COVID-19-vaccines till today, we believe that such isolated events should not challenge the policy supporting widespread vaccination to fight COVID-19-pandemic.

## Data Availability

Not applicable.

## Abbreviations

CSF: Cerebrospinal fluid
CT: Computed tomography
CN: Cranial nerve
LN: Lymph node
HF: High-frequency
MRI: Magnetic resonance imaging
HHV: Human herpesvirus
